# Obstetric Management of a Patient with Narcolepsy and Cataplexy: A Case Report

**DOI:** 10.1155/2012/982039

**Published:** 2012-08-13

**Authors:** S. Ajayi, R. Kinagi, E. Haslett

**Affiliations:** Department of Obstetrics & Gynaecology, Blackpool Teaching Hospitals, Whinney Heys Road, Blackpool, Lancashire FY3 8NR, UK

## Abstract

We report the management of a patient who suffers from narcolepsy and cataplexy and presented to the clinic at 14 weeks of gestation. Her symptoms were resistant to modafinil but controlled with clomipramine and amphetamine. Discussion concerning mode of delivery for these patients is scarce in the literature. We discuss some issues surrounding the antenatal management and counselling regarding mode of delivery and postpartum care.

## 1. Introduction

Narcolepsy is a chronic neurological condition producing disruption to normal sleep pattern resulting into excessive daytime somnolence. Approximately 80% of the patients with narcolepsy suffer from cataplexy (involuntary loss of muscle tone) [1], and cataplexy is pathognomonic for narcolepsy. There is little information on outcome or management of this disorder in pregnancy. We report a case of successful management of pregnancy in a patient with narcolepsy and cataplexy.

## 2. Case Report

A 44-year-old G3 Para1 (previous normal vaginal delivery at term 24 years ago) with a past history of narcolepsy and cataplexy was referred to the combined medical/obstetrics antenatal clinic at 14 weeks of gestation.

Narcolepsy and cataplexy was diagnosed in January 2005 and since then had been under regular Neurological review. She had developed daytime sleepiness, cataplexy, sleep paralysis, and hallucination on going to sleep. Cataplexy was controlled with clomipramine, and amphetamine (dexamphetamine) was started in November 2010 when her symptoms worsened. Her symptoms were resistant to modafinil.

Antenatally the patient was managed in conjunction with the neurologist, and referral was made to the anaesthetist, and a paediatric alert was made in view of amphetamine use in early pregnancy. The pregnancy proceeded uneventfully, and the dose of dexamphetamine was reduced during the pregnancy and eventually discontinued at 24 weeks of gestation.

She was reviewed by the anaesthetist at 36 weeks of gestation due to concerns regarding option of analgesia and mode of delivery if she presents in labour. She was also reviewed by the consultant obstetrician and after weighing the benefits and risks of elective delivery versus spontaneous labour opted for an elective Caesarean section at nearly 38 weeks of gestation with steroid cover as per unit protocol.

The Caesarean section was performed under spinal analgesia and was uneventful. A female baby weighing 2.84 Kg was delivered with Apgar score 7, 10, and 10 at 1st, 5th, and 10th minutes of life. Cord gases were pH (V) 7.383 BE-2.8, pH (A) 7.361 BE-6.1.

Baby needed only facial oxygen at birth. Neonatal abstinence syndrome (NAS) scoring was not required for baby.

## 3. Discussion

Narcolepsy is a chronic neurological condition producing disruption to normal sleep pattern, and this causes excessive daytime somnolence. Approximately 80% of patients with narcolepsy suffer from cataplexy (involuntary loss of muscle tone) [1], and cataplexy is pathognomonic for narcolepsy. The cause of narcolepsy is unknown, but it is thought that both environmental and genetic factors may play apart. It may be caused by the loss of a relatively few neurons that are responsible for producing hypocretin-a neuropeptide in the CNS. It is also associated with specific HLA allele, DQB1∗0602. Possible triggers include head trauma, infection, and change in sleeping habits [2]. Diagnostic criteria including excessive daytime sleepiness, almost daily for greater than 3 months, and cataplexy triggered by emotion, confirmed by diagnosis with nocturnal polysomnography followed by a multiple sleep latency test (MSLT) and alternatively confirmed by CSF hypocretin-1 levels <110 pg/mL or one-third of mean control values [3].

Patient with narcolepsy should be referred to a sleep disorder service and managed by a clinician with experience of narcolepsy management. This involves largely symptom relief with nondrug treatment such as good sleep hygiene, daytime naps, regular exercise, and patient's/family education. Drug therapy includes use of stimulant such as amphetamines and methylphenidate (2nd line), modafinil (1st line), antidepressants, or SSRIs (selective serotonin reuptake inhibitors) to improve symptoms of sleep paralysis or hypnagogic hallucination. Benzodiazepines may be used sometimes to consolidate nighttime sleep in refractory cases [4]. It was noticed that our patient's symptoms were resistant to modafinil (none reported in the literature to date). Cataplexy can be managed with sodium oxybate and antidepressants such as clomipramine, SSRIs (venlafaxine), and selegiline [5]. Most of these drugs have significant adverse effects and should be used under specialist recommendation.

It is worth noting that there is little guidance as to the management of this condition in pregnancy as well as the mode of delivery with only two reports in the literature of pregnant narcoleptics with cataplexy undergoing Caesarean delivery.

Due to the risk of labour-induced cataplexy, a pregnant woman with narcolepsy and clinically significant cataplexy should discuss the option of elective Caesarean delivery with the physician and obstetrician [6]. Regional anaesthesia including neuraxial opioids for Caesarean section is a suitable technique in patients with narcolepsy [1].

After delivery, safety precautions are indicated to enhance safety of the mother and the new born if no medication was used during pregnancy or continued in the postnatal period. This is as a result of the exacerbation excessive daytime sleepiness (EDS) in the narcoleptics caused by the physical demands and sleep deprivation of childcare [6]. If medication were used in pregnancy, a neonatal abstinence syndrome (NAS) would be required. Babies presenting with symptoms such as high-pitched cry, tremor, jittery, irritability, and sleeping difficulty should be assessed. When there is a combination of these symptoms, a diagnosis of NAS is made, and appropriate review and management by the paediatrician would be required [7].

On discharge from the hospital, mothers should watch out for symptoms such as difficulty with attachment during breastfeeding, colic, poor sleeping pattern, and slow weight gain. The importance of safe sleeping should also be discussed and appropriate support offered [7].

In summary, we have presented a successful management of a patient with narcolepsy and cataplexy in pregnancy. This was achieved with good multidisciplinary care involving the obstetrician, neurologist, anaesthetist, paediatrician, and midwife with each professional contributing to patient's care.
